# The Identification and Characterization of Two Novel Epitopes on the Nucleocapsid Protein of the Porcine Epidemic Diarrhea Virus

**DOI:** 10.1038/srep39010

**Published:** 2016-12-19

**Authors:** Kang Wang, Chun Xie, Jianan Zhang, Wenchao Zhang, Deqiang Yang, Lingxue Yu, Yifeng Jiang, Shen Yang, Fei Gao, Zhibiao Yang, Yanjun Zhou, Guangzhi Tong

**Affiliations:** 1 Department of Swine Infectious Diseases, Shanghai Veterinary Research Institute, Chinese Academy of Agricultural Sciences, Shanghai, 200241, P. R. China; 2 Shanghai Key laboratory of Veterinary Biotechnology, School of Agriculture and Biology, Shanghai JiaoTong University, Shanghai, 200240, P. R. China; 3 Jiangsu Co-innovation Center for Prevention and Control of Important Animal Infectious Diseases and Zoonoses, Yangzhou, 225009, P. R. China

## Abstract

Porcine epidemic diarrhea virus (PEDV) is a highly contagious *coronavirus* that causes severe diarrhea and death, particularly in neonatal piglets. The nucleocapsid protein (N protein) of PEDV presents strong immunogenicity and contributes to the cross-reactivity between PEDV and TGEV. However, the characterization of epitopes on the PEDV N protein remains largely unknown. Here, two monoclonal antibodies (MAbs) specific to the N protein of a PEDV strain, FJzz1/2011, were generated and screened against a partially overlapping library of 24 GST-fusion N protein-truncated constructs. We confirmed that residues 18–133 (designated NEP-D4) and residues 252–262 (designated NEP-D6) were the epitopes targeted by MAbs PN-D4 and PN-D6, respectively. Sequence analysis revealed that these two epitopes were highly conserved among PEDV strains but were significantly different from other members of the *Coronavirinae* subfamily. Western blot analysis showed that they could be specifically recognized by PEDV antisera but could not be recognized by TGEV hyperimmune antisera. Indirect immunofluorescence (IFA) assays confirmed no cross-reaction between these two MAbs and TGEV. In addition, the freeze-thaw cycle and protease treatment results indicated that NEP-D4 was intrinsically disordered. All these results suggest that these two novel epitopes and their cognate MAbs could serve as the basis for the development of precise diagnostic assays for PEDV.

Porcine epidemic diarrhea virus (PEDV) is a member of the genus *Alphacoronavirus*, which belongs to the family *Coronaviridae* in the order *Nidovirales*. The genome of this single-stranded, positive-sense RNA virus encodes, from 5′ to 3′, the replicase polyproteins pp1a and pp1b, spike (S) protein, ORF3 accessory protein, envelope (E) protein, membrane (M) protein and nucleocapsid (N) protein^1^,^2^,^3^. The typical clinical signs of PEDV infection include watery diarrhea, vomiting, and dehydration. Porcine epidemic diarrhea (PED) was first reported in England in 1971 and then in many other European and Asian countries in the following 30 years. With the advent of live attenuated vaccines, acute outbreaks became rare in both Europe and Asia during 2000–2006^4^,^5^,^6^,^7^,^8^. Since 2007, however, PEDV has re-emerged and spread across the globe, reaching Asia (Thailand, Vietnam, Japan, and China), Europe (Germany, Spain, Belgium, France, and Portugal), North America (US, Mexico, and Canada), and Australia. These outbreaks were characterized by high morbidity (80–100%) and mortality (50–90%) among suckling piglets^9^,^10^,^11^,^12^,^13^,^14^,^15^,^16^,^17^,^18^,^19^,^20^,^21^,^22^,^23^, causing substantial financial losses to the domestic swine industry. The severity of these epidemics emphasizes the importance of establishing effective disease control and prevention systems worldwide^24^, which will largely depend on well-validated diagnostics tests and vaccinations for PEDV.

The coronavirus N protein binds viral RNA and is involved in several of the biological activities of the virus^25^,^26^. Previous studies have shown that PEDV N protein is responsible for viral nucleolar localization, host cycle ER stress, S-phase prolongation, up-regulation of interleukin-8 expression and inhibition of interferon-β production^27^,^28^,^29^. In addition, PEDV N protein is highly conserved. Large amounts of PEDV N protein can be detected in virus-infected cells, and abundant antibodies against N protein could be induced at the early stage of PEDV infection^30^,^31^. Such features of the N protein make it an ideal target antigen for PEDV diagnosis^32^, emphasizing the importance of characterizing the epitopes of PEDV N protein and their related MAbs. Previous research on this issue has been scarce. To our knowledge, only one MAb specific to PEDV N protein has been reported so far, with little information regarding its corresponding epitope and whether it could cross-react with TGEV N protein^33^. More detailed research aimed at characterizing PEDV N protein epitopes is urgently needed.

Acute diarrhea caused by PEDV is clinically difficult to distinguish from disease caused by transmissible gastroenteritis virus (TGEV). Therefore, PEDV infection cannot be diagnosed on the basis of clinical findings alone, and laboratory tests are necessary for an etiological diagnosis^34^. Presently, the most commonly used diagnostic methods are reverse transcription-polymerase chain reaction (RT-PCR) and real-time PCR, both of which have a high efficiency and reliability in detecting viral genetic materials in fecal and intestinal samples. In addition, immunofluorescence assays (IFAs), electron microscopy (EM), immunohistochemistry, *in situ* hybridization, enzyme-linked immunosorbent assays (ELISAs), immunochromatography assays and fluorescent microsphere immunoassays (FMIAs) have been widely used to diagnose PEDV infection^35^,^36^,^37^,^38^,^39^,^40^,^41^,^42^,^43^,^44^,^45^. Among these methods, ELISA has the greatest potential for clinical diagnosis on a large scale and the greatest significance for the effective detection and control of PEDV, especially in developing countries, where few pig farms are equipped with qualified laboratories for complicated molecular diagnoses. However, the previously established method of indirect ELISA used either the viral proteins extracted from PEDV-infected Vero cells or recombinant N protein generated in *Escherichia coli* as coating antigens; in some documented DAS-ELISA methods, the specificity of the capture and detection antibodies was unclear^40^,^41^,^46^. Furthermore, in a recent study, researchers observed the cross-reactivity between PEDV and pig TGEV antisera. They also inferred that one or more epitopes on the N protein could be the contributing factors^47^. These findings called into question the accuracy of current ELISA methods. To establish more reliable serodiagnostic methods for PEDV, it is important to generate MAbs that bind only to PEDV N protein and to map which of their corresponding epitopes that can be recognized by PEDV pig antisera alone.

Most proteins carry out biological functions based on their unique and stable three-dimensional structures. However, studies in recent decades have reported an increasing number of proteins that are fully (intrinsically disordered proteins, IDPs) or partially (intrinsically disordered regions, IDRs) unfolded or disordered under normal physiological conditions yet remain active and functional^48^,^49^,^50^,^51^. Moreover, the essential roles of IDPs/IDRs in the protein interaction network, including cell signaling, transcription, translation, and the cell cycle, have been confirmed^50^,^52^,^53^,^54^. In particular, disorder abundance can be predicted and observed in viral proteins^55^,^56^. The unstable structures of IDPs/IDRs make it difficult for the immune system to recognize the viral epitopes, eliciting a poor immune response from the host. Partially or wholly unfolded viral proteins can also quickly adapt to and survive in the host environment due to the very high mutation rates of viral genomes^57^,^58^. Moreover, IDPs, as important components of antigens, are abundant in several pathogenic organisms^59^,^60^. Disordered epitopes are distinct and specific, and they efficiently interact with their cognate antibodies^60^,^61^. Studies have demonstrated that the molecular mechanism by which IDPs function is closely associated with the transition from the disordered to the folded state upon binding with their partners, which could be proteins, DNA, RNA or membranes^51^,^62^,^63^. However, a similar phenomenon in terms of the recognition of disordered epitopes by antibodies has not yet been reported. Studies on SARS have demonstrated that IDRs comprise the flexible linker between the N-terminal domain (NTD) and C-terminal domain (CTD) in N protein and have RNA-binding abilities^64^. However, little attention has been paid to characterizing other intrinsically disordered epitopes of Coronaviruses.

To better understand the characterization of epitopes on PEDV N protein, in this study, two MAbs against N protein (PN-D4 and PN-D6) were generated, and their corresponding epitopes (NEP-D4 and NEP-D6) were identified and characterized. Further analysis based on sequence alignment, IFA and Western blotting showed the conservation, specificity and immunogenicity of NEP-D4 and NEP-D6. We also demonstrated that both NEP-D4 and NEP-D6 failed to result in cross-reactivity between PEDV and TGEV. Thus, these two MAbs (PN-D4 and PN-D6) and their corresponding epitopes (NEP-D4 and NEP-D6) could be theoretically applied to clinically accessible diagnostic assays for PEDV. In addition, GeneSilico metadisorder and Phyre2 3D structure prediction, freeze-thaw cycle analysis, and treatment with trypsin and DTT all indicated that NEP-D4 is probably an intrinsically disordered protein. Based on these findings, we speculated that the folding following the binding effect might be the factor behind the recognition of NEP-D4 by PN-D4. This finding has not been previously reported but might warrant further investigation aimed at better understanding the mechanism by which a disordered epitope combines with its cognate antibody.

## Results

### Production and Selection of MAbs against PEDV N protein

**Figure 1:**
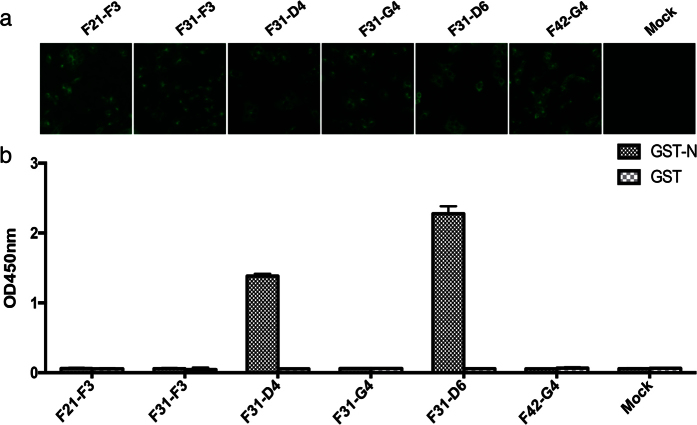
Selection of MAbs against PEDV N protein. (**a**) Six MAbs were proven to be positive by IFA using PEDV inoculated cells. (**b**) The six positive clones were then tested by ELISA against GST-fusion N protein and GST-tag expressed in *Escherichia coli*.

To generate MAbs specific to the PEDV N protein, we immunized BALB/c mice with the purified PEDV strain FJzz1/2011. We acquired hybridoma cells by fusing SP20 cells and the spleen cells from immunized mice. Next, the hybridoma cell lines that survived culture in HAT and HT media were screened by IFA against the PEDV strain FJzz1/2011. We obtained six positive clones (F21-F3, F31-F3, F31-D4, F31-G4, F31-D6 and F42-G4) that were specific to PEDV (Fig. 1a). We then examined which MAbs could detect the GST-tagged recombinant N protein using indirect ELISA. F31-D4 and F31-D6 exhibited a positive color, whereas F21-F3, F31-F3, F31-G4 and F42-G4 were negative (Fig. 1b). We designated the MAbs secreted by hybridomas F31-D4 and F31-D6 as PN-D4 and PN-D6, respectively. The results suggested that MAbs PN-D4 and PN-D6 are specific to PEDV N protein.

### Fine epitope mapping of PEDV N protein

**Figure 2:**
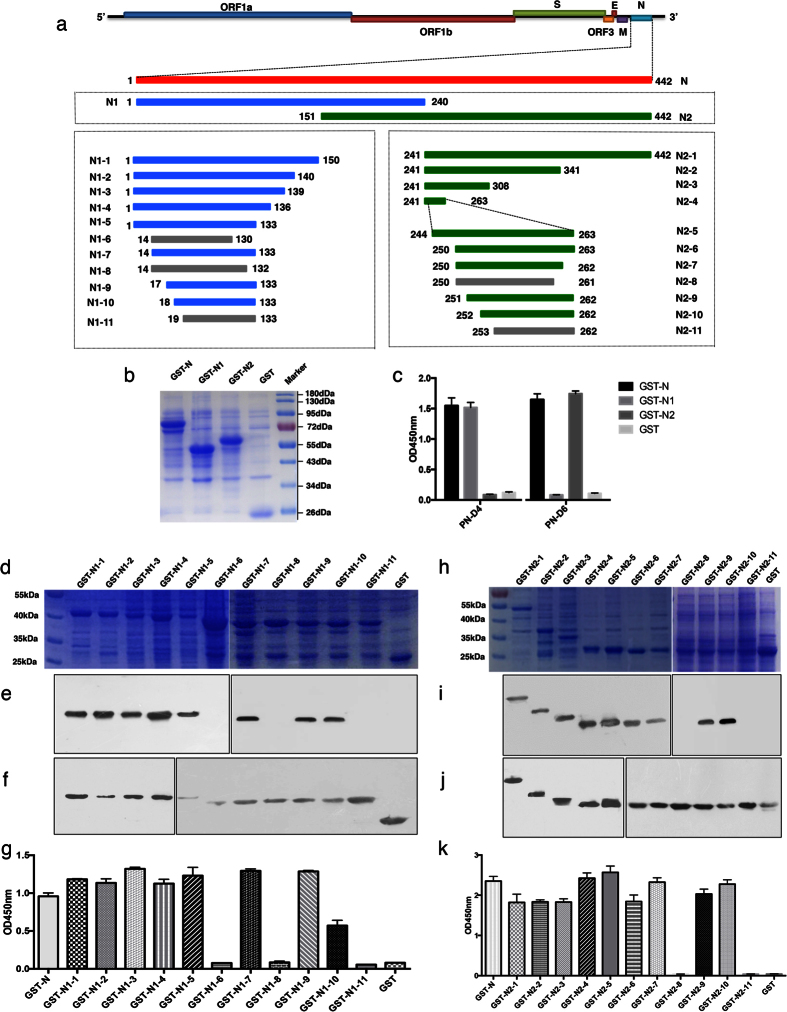
Mapping of PEDV N protein epitopes. (**a**) Schematic diagram of the epitope mapping. The segments that could be recognized by both PN-D4 and PN-D6 are highlighted in red; the segments that could only be recognized by PN-D4 are highlighted in blue; the segments that could only be recognized by PN-D6 are highlighted in green; and the segments in gray are those that could not be recognized by either PN-D4 or PN-D6. According to (**a**), the N gene was divided into mutually overlapping N1 and N2 fragments and was directionally cloned into the pGEX-6p-1 vector. (**b**) Six hours after the addition of 1 mM IPTG, the induced products of GST-N, GST-N1 and GST-N2 were processed and analyzed using SDS-PAGE (12% separating gel and 5% stacking gel). (**c**) Next, the GST fusion proteins GST-N, GST-N1 and GST-N2 were coated onto ELISA plates (0.5 μg/well) and were probed with MAbs PN-D4 and PN-D6. After the first round of identification, N1 and N2 were further divided and expressed in two series of fusion proteins, GST-N1-1-GST-N1-11 and GST-N2-1-GST-N2-11 (**d**,**h**), which were analyzed by Western blotting and ELISA using PN-D4 (**e**,**g**), PN-D6 (**i**,**k**), and anti-GST Tag antibody (**f**,**j**), respectively.

**Figure 3:**
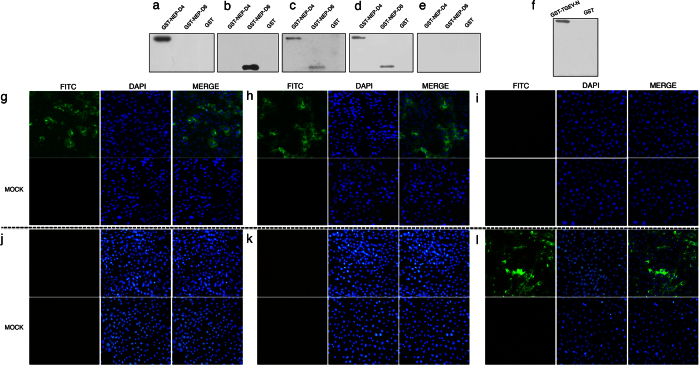
The reactivity between epitopes and MAbs or antisera. The two fusion-expressed epitopes (GST-NEP-D4 and GST-NEP-D6) and the negative control (GST tag) were processed and analyzed by Western blotting using PN-D4, PN-D6, or pig PEDV antisera obtained at 14 and 21 days after starting the immunization. Pig TGEV hyperimmune antisera was used as the primary antibodies in membranes **a**,**b**,**c**,**d** and **e**. (**f**) We used the pig TGEV hyperimmune antisera to test GST-TGEV-N protein and GST tag. In IFA, monolayers of Vero cells inoculated with the PEDV strain HLJ/2011 or FJzz1/2011 and ST cells inoculated with the TGEV strain SH/2012 were fixed for IFA against MAbs PN-D4, PN-D6 or anti-TGEV N protein. (**g**) The PEDV strain HLJ/2011 was detected by MAb PN-D4. (**h**) The PEDV strain HLJ/2011 was detected by MAb PN-D6. (**j**) The TGEV strain SH/2012 was detected by MAb PN-D4. (**k**) The TGEV strain SH/2012 was detected by MAb PN-D6. (**i**) The PEDV strain FJzz1/2011 was detected by the MAb anti-TGEV N protein. (**l**) the TGEV strain SH/2012 was detected by the anti-TGEV N protein.

To identify the antigenic determinants that could be recognized by MAbs PN-D4 and PN-D6, two mutually overlapping GST-fusion-truncated constructs (GST-N1 and GST-N2) that span the complete N protein were expressed for detection by MAbs using ELISA (Fig. 2b and c). MAb PN-D4 recognized only GST-N1, whereas MAb PN-D6 only recognized GST-N2 (Fig. 2c). To locate the precise positions of the two epitopes, as depicted in Fig. 2a, N1 and N2 were further truncated into two discrete groups of GST-fusion constructs, which were detected by PN-D4 or PN-D6 in ELISA and Western blotting assays. The results in Fig. 3 showed that the minimum antigenic epitopes that MAbs PN-D4 and PN-D6 could recognize were N1-10 (residues 18–133) and N2-10 (residues 252–262), respectively, which were designated NEP-D4 and NEP-D6, respectively.

### Immunogenicity analysis of the epitopes

To determine whether PEDV-infected pigs could produce specific antibodies against NEP-D4 and NEP-D6, we performed Western blotting using anti-PEDV porcine antisera obtained 14 and 21 days after immunization to detect the two GST-fusion peptides. The results showed that both lanes loaded with NEP-D4 or NEP-D6 produced positive bands at the expected molecular masses, indicating that NEP-D4 and NEP-D6 are both immunogenic epitopes of PEDV.

### The epitopes are highly conserved and specific

**Figure 4:**
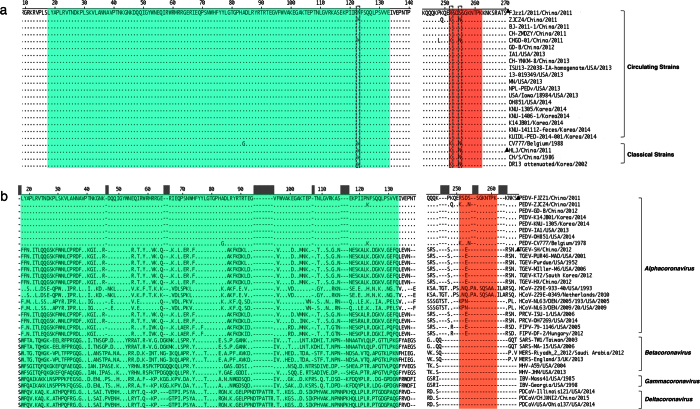
Amino acid sequence analysis of the two identified epitopes among different virus strains. (**a**) Amino acid sequence alignments of NEP-D4 and NEP-D6 from 23 PEDV representative strains, including 4 classical strains and 19 currently circulating strains. (**b**) Similarly, residues 18–133 and residues 252–262 of 21 *Alphacoronavirus* strains, including seven PEDV strains, six TGEV strains, two human coronavirus 229E strains, two human coronavirus NL63 strains, two PRCV (porcine respiratory coronavirus) strains and two FIPV (feline infectious peritonitis virus) strains, 6 *Betacoronavirus* strains, including two SARS-CoV (severe acute respiratory syndrome coronavirus) strains, two MERS-CoV (Middle East respiratory syndrome coronavirus) strains and two MHV (mouse hepatitis virus) strains, 2 IBV (Infectious bronchitis virus) strains from *Gammacoronavirus*, and 3 PDCoV (porcine deltacoronavirus) strains from *Deltacoronavirus*, were selected for alignment. The corresponding antigenic positions of NEP-D4 and NEP-D6 of all strains are colored green and red, respectively. PEDV strains FJzz1/2011 and HLJ/2011 isolated from our laboratory were marked with black triangles. The major mutations are highlighted by dotted frames.

To investigate the conservation of the determined epitopes NEP-D4 and NEP-D6 among different classical and presently circulating PEDV strains, 21 representative strains from GenBank and one strain isolated in our laboratory, HLJ/2011, were selected for sequence alignment using DNASTAR. The result showed that the sequence similarities of NEP-D4 and NEP-D6 among different PEDV strains were 98.3–100% and 81.8–100%, respectively, indicating that NEP-D4 and NEP-D6 were conserved. However, three conspicuous amino acid substitutions, including Asn^123^ to Lys in NEP-D4 and Arg^252^ to Lys and Ser^255^ to Asn in NEP-D6, were observed in several particular strains (Fig. 4a).

To further test whether these mutations changed the antibody-antigen interactions, we used MAbs PN-D4 and PN-D6 to detect the PEDV strain HLJ/2011 by IFA. HLJ/2011 contains all of these mutations and can be stably passaged in Vero cells. The results revealed that HLJ/2011 could be recognized by both PN-D4 and PN-D6 (Fig. 3g and h), suggesting these three substitutions do not alter the antigenicity of epitopes NEP-D4 and NEP-D6 and that both NEP-D4 and NEP-D6 are highly conserved. In addition, sequence alignment among all the known members within subfamily *Coronavirinae* revealed that epitope NEP-D4 and NEP-D6 are highly specific to PEDV (Fig. 4b) because the homology of these two regions between PEDV and all other members of *Coronavirinae* remains very low (<44%).

### Cross-reactivity analysis

To investigate whether PEDV cross-reacts with TGEV on the epitopes NEP-D4 and NEP-D6, we performed IFA against the TGEV strain SH/2012 using MAbs PN-D4 or PN-D6 as the primary antibody. No fluorescent signals were observed (Fig. 3j and k). Moreover, in Western blotting assays, no bands appeared to indicate the interaction of TGEV hyperimmune antisera and the epitope NEP-D4 and NEP-D6 (Fig. 3e). These results suggested that the epitopes NEP-D4 and NEP-D6 could not cause cross-reactivity between PEDV and TGEV.

### Intrinsically disordered regions on epitope NEP-D4

**Figure 5:**
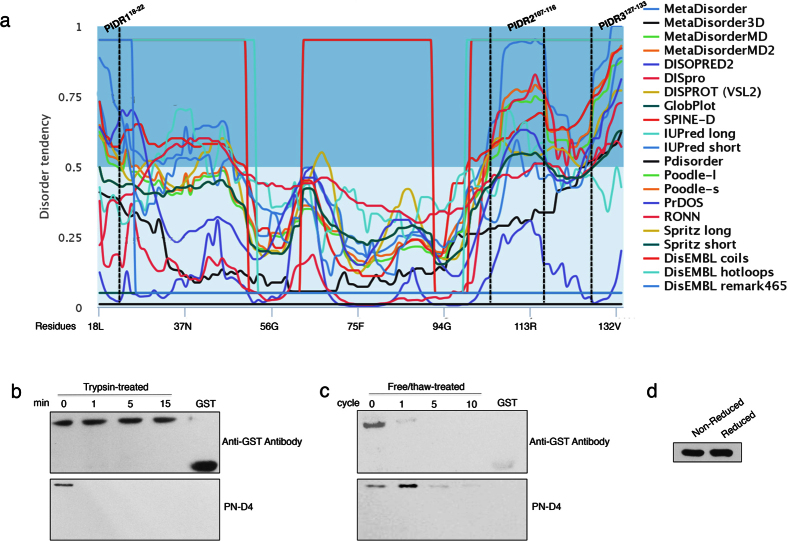
Identification of IDRs and disulfide bridges in epitope NEP-D4. (**a**) The disorder plot for epitope EP4 was predicted by GeneSilico metadisorder. The x-axis shows the residues from 18 to 133, and the y-axis shows the disordered tendency ranging from 0 to 1. All residues with a disorder probability of more than 0.5 were considered to be disordered. The residues with plotted lines in the light blue region were predicted to be disordered; their counterparts in the bluish white area were considered ordered. (**b**) Four GST-NEP-D4 samples were treated with Trypsin under the same conditions, and the reactions were stopped at 0 min, 1 min, 5 min and 15 min. Next, the samples were processed and analyzed by Western Blotting using anti-GST antibody and PN-D4 as the primary antibodies. (**c**) Similarly, four additional GST-NEP-D4 samples were treated with freeze (−80 °C) and thaw (room temperature) cycles, which were repeated up to 0, 1, 5 and 10, times, respectively. Next, these samples were analyzed by Western blotting using the same antibodies above. (**d**) To rule out the interference of disulfide bridges, the reduced (1 M DTT-treated) and non-reduced (non DTT-treated) GST-NEP-D4 were analyzed by Western blotting using PN-D4.

To better understand the effect of NEP-D4 in combination with PN-D4, we used multiple online tools to compute the physical and chemical parameters of NEP-D4. One of these tools, the GeneSilico MetaDisorder server, was used to predict the intrinsic disorder. Three regions presented with the highest propensity for intrinsic disorder, which we designated as PIDR^18^,^19^,^20^,^21^,^22^, PIDR^107–116^ and PIDR^127–133^ (Fig. 5a). The prediction suggested that there might be some intrinsically disordered regions on NEP-D4. To verify this prediction, purified GST-tagged NEP-D4 was treated with trypsin and used for detection against PN-D4 or anti-GST antibody by Western blotting. We observed that the trypsin-treated peptide was recognized by anti-GST antibody but not by MAb PN-D4 (Fig. 5b). In addition, we repeated the experiment with the freeze-thaw cycle-treated GST-tagged NEP-D4. The result showed that the treated peptide (up to 5 and 10 cycles) could be recognized by MAb PN-D4 but not by the anti-GST antibody (Fig. 5c). The intrinsically disordered proteins have a high proportion of solvent-exposed residues, making them highly accessible to protease and resistant to cold treatment^65^,^66^. Taken together, these results suggested that NEP-D4 is likely to be an intrinsically disordered protein.

### 3D structure modeling of the epitopes

**Figure 6:**
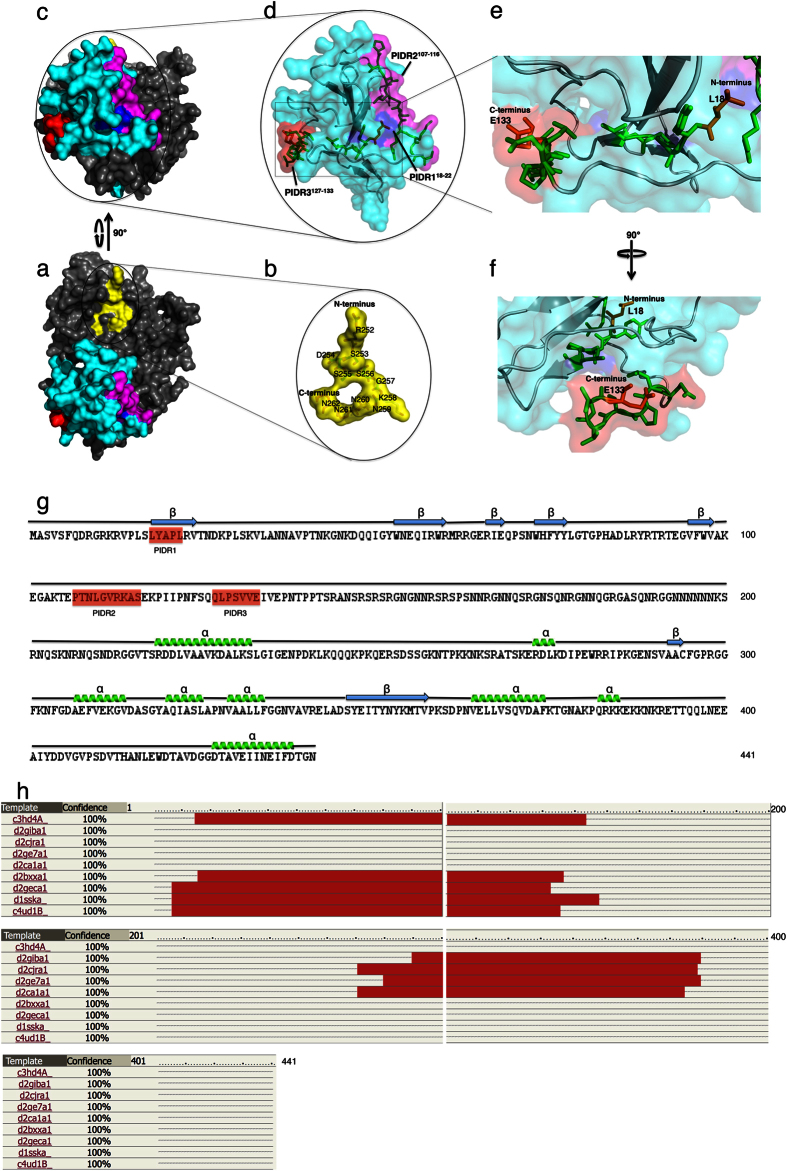
Predicted 3D structural model of N protein with Phyre2. The N protein 3D model was visualized using the PyMOL molecular graphics and modeling system and was viewed from the side (**a**) and top (**c**). The protein epitope domains identified in this study are highlighted in different colors. (**b**) The conformation of the epitope NEP-D6 is displayed with all residues indicated in the corresponding positions. (**d**) The secondary structure of epitope NEP-D4 is depicted, in which the predicted disordered regions are colored green and tertiary structures showing PIDR1^18^,^19^,^20^,^21^,^22^, PIDR2^107–116^ and PIDR3^127–133^ are colored blue, magenta and red, respectively. (**e**,**f**) The putative spatial positions of critical residues, E^133^ and L^18^, which are represented by orange. (**g**) The sequence of N protein with alpha helixes, beta strands and PIDRs. (**h**) Template coverage used in the structure modeling.

To clarify the spatial locations of NEP-D4 and NEP-D6 on N protein, we generated a 3-dimensional structural model of PEDV N protein using intensive modeling based on nine templates in the Phyre2 server. The prediction result showed that 251 of 441 residues (57%) were modeled at >90% accuracy; the remaining 191 residues were modeled *ab initio*. The amino acids of NEP-D6 were located in close proximity and were exposed on the surface of the N protein, suggesting that NEP-D6 is highly likely to be a linear epitope. By contrast, NEP-D4 is much more complex; although most of the amino acids are located on the surface, a small fraction of its residues are buried inside the N protein, suggesting that NEP-D4 is more likely to be a conformational epitope (Fig. 6).

## Discussion

As one of the most serious intestinal infectious diseases, PED has posed a substantial threat to the swine industry of many countries over the last few years^12^,^16^,^67^. The accurate diagnosis at the early stage of infection plays a key role in PEDV prevention. The N protein of PEDV consists of 441 amino acids and represents an essential agent in the formation of the virus nucleocapsid structure. The abundant expression of N protein in PEDV-infected cells and the high conservation among different PEDV strains make it fairly suitable for use as a target for PEDV diagnosis^30^,^68^. However, the epitopes on PEDV N protein have been poorly characterized. In the present study, six monoclonal antibodies were generated against PEDV from BALB/c mice immunized with the purified PEDV strain FJzz1/2011. Among these antibodies, MAbs PN-D4 and PN-D6 were demonstrated as N protein-specific; the remaining four MAbs are still being studied (data not shown). Combined with ELISA and Western blotting, peptide scanning was performed to identify the epitopes of MAbs PN-D4 and PN-D6. We confirmed that Leu^18^ and Ile^133^ were the essential amino acids of the epitope NEP-D4 because the deletion of either of these amino acids stopped the recognition between NEP-D4 and PN-D4. Notably, the absorbance value also largely declined when the peptide was truncated at Leu^18^ instead of Ser^17^. Therefore, Ser^17^ might have a significant effect on the antigenicity of NEP-D4, although Leu^18^ was the only essential amino acid at the N-terminus of this epitope. Likewise, Arg^252^ and Lys^262^ are crucial amino acids for the binding of epitope NEP-D6 with MAb PN-D6. The results suggested that NEP-D4 (residues 18–133) and NEP-D6 (residues 252–262) are the specific antigenic epitopes of MAbs PN-D4 and PN-D6, respectively.

The epitopes NEP-D4 and NEP-D6 steadily exist on different PEDV strains that are both classical and currently circulating (Fig. 4a). Additionally, the sequence similarities of the epitopes NEP-D4 and NEP-D6 with the representative strains were 98.3–100% and 81.8–100%, respectively. Although three major mutations, Asn^123^ to Lys, Arg^252^ to Lys, and Ser^255^ to Asn, were observed in certain strains, these substitutions do not alter the antigenicity of NEP-D4 and NEP-D6 because PN-D4 and PN-D6 could precisely recognize another PEDV strain, HLJ/2011, which was isolated in our laboratory and contains all three of these mutations (Fig. 3g and h). In addition, we demonstrated that NEP-D4 and NEP-D6 could be recognized by PEDV pig antisera collected at 14 and 21 days after inoculation with PEDV, indicating that PEDV infection could induce specific host antibodies against NEP-D4 and NEP-D6 (Fig. 3c and d). These findings suggested that epitopes NEP-D4 and NEP-D6 were highly conserved and immunogenic; both MAbs PN-D4 and PN-D6 and epitopes NEP-D4 and NEP-D6 have the potential for use as effective tools in the serological diagnosis of PEDV during early stages of infection. Thus far, N protein and MAbs against N protein have been widely used for the diagnosis of PEDV. Hou *et al*. established an ELISA system using recombinant N protein generated in *Escherichia coli*^69^; Okda *et al*. established indirect ELISA and FMIA (Fluorescent microsphere immunoassay) based on recombinant N protein, and blocking ELISA based on N protein-specific MAbs^45^.

Recently, a one-way cross-reactivity between PEDV strains and TGEV hyperimmune pig antisera was clearly observed, and one or more epitopes on the N-terminal region of N protein were hypothesized to be the root cause of this cross-reactivity^45^. Thus, the epitopes of N protein used for PEDV diagnosis should be highly PEDV-specific and the reported immunoassays above should be seriously re-examined. We analyzed the NEP-D4 and NEP-D6 epitope sequences of PEDV and other members within the subfamily *Coronavirinae*, especially TGEV, PRCV, and PDCoV, all of which can infect newborn piglets. We found that the amino acid sequences of NEP-D4 and NEP-D6 of PEDV differed greatly from their counterparts in other coronaviruses. NEP-D4 shared less than 43.1% homology with TGEV and PRCV of *Alphacoronavirus*, and only 26.5% homology with PDCoV of *Deltacoronavirus*. The homology of NEP-D6 between PEDV and the other three viruses (TGEV, PRCV, and PDCoV) remained at zero. We also used IFA to investigate whether NEP-D4 and NEP-D6 could induce cross-reactivity between PEDV and TGEV. The results showed that neither PN-D4 nor PN-D6 recognized TGEV, and the TGEV SH/2012 hyperimmune pig antisera failed to react with NEP-D4 and NEP-D6; these results indicated that NEP-D4 and NEP-D6 were unable to cause a cross-reaction between PEDV and TGEV. Therefore, the MAbs PN-D4 and PN-D6 and their corresponding epitopes NEP-D4 and NEP-D6 have the potential for use in developing an immunoassay that differentiates between PEDV and TGEV.

The epitope NEP-D6 has 11 amino acids and can be recognized by PN-D6 using Western blotting, a method that imposes protein denaturation. This finding indicates that NEP-D6 is a linear epitope, which agreed with the result that NEP-D6 is located on the surface of the predicted 3D structure of N protein (Fig. 6a and b). However, the epitope NEP-D4 seems much more complicated. Western blotting suggested that NEP-D4 is a linear epitope, which contradicts the previous assumption that the full 116 aa length is too large to be a linear epitope when bound to an antibody. Again, multiple residues are buried inside N protein, and there is a significant spatial distance between the first residue (Leu^18^) and last residue (Glu^133^) of NEP-D4 (Fig. 6d,e and f). The NEP-D4 portion of the 3D model is highly reliable because the residues 18–133 were constructed based on five templates (PDB IDs: c3hd4A, d2bxxa1, d2geca1, d1sska and c4ud1B) at nearly 100% confidence, as shown in Fig. 6h. Therefore, NEP-D4 is more likely to be a conformational epitope than a linear epitope. Interestingly, a denatured conformational epitope is unexpectedly recognized by its cognate antibody in Western blotting. Although it is possible that the formation of some disulfide bridges between certain residues may have contributed to this binding effect, this possibility was ruled out by the ability of PN-D4 to react with dithiothreitol (DTT)-treated NEP-D4 in a Western blotting assay (Fig. 5d). Additionally, no disulfide bridges were predicted by the online software PredictProtein (data not shown). We also analyzed the other properties of NEP-D4. The software GeneSilico metadisorder predicted that NEP-D4 is an intrinsically disordered protein, which was further confirmed by two independent experiments: namely, trypsin-treated NEP-D4 could not react with PN-D4, and NEP-D4 underwent freeze-thaw cycles (up to 5 and 10 times) that were indeed recognized by PN-D4, albeit weakly. As previous findings have shown, the solvent-exposed residues on IDPs make proteins highly susceptible to protease; moreover, IDPs have much higher cold stability than structured proteins^65^,^66^. Thus, we speculated that folding occurred upon binding, which may have led to the recognition of NEP-D4 by PN-D4. Specifically, when the antibody binds to the first or last residue of the epitope NEP-D4, it may trigger the disordered domain to fold completely to form a certain conformational site, which could, in turn, be recognized by MAb PN-D4. In a previous study, different PEDV and TGEV pig antisera were used in a two-way cross-reactivity examination of different PEDV and TGEV strains, but only a one-way reactivity between TGEV Miller hyperimmune pig antisera and PEDV strains was observed. The complex mechanism remains unclear^47^. However, given that the N-terminal region of the N protein is intrinsically disordered, some disordered epitopes such as NEP-D4, which may be folded under certain conditions, might be the cause of the one-cross reactivity between PEDV and TGEV. In other words, the antibodies generated from PEDF infection cannot induce the folding of the disordered epitope on TGEV, whereas the corresponding epitope on PEDV can fold upon the binding of the antibodies produced from the infection of certain TGEV strains with specific epitopes. These hypotheses merit further investigation.

In conclusion, we first identified two highly conserved and specific epitopes on the N protein of PEDV. While NEP-D6 (residues 252–262) is a linear epitope, NEP-D4 (residues 18–133) is an intrinsically disordered epitope. The recognition between NEP-D4 and its cognate antibody might require folding effects upon antibody binding. Most importantly, epitopes NEP-D4 and NEP-D6 and MAbs PN-D4 and PN-D6 could be used as very effective tools in diagnosing PEDV, even at very early stages of PEDV infection.

## Methods

### Ethics Statement

All animal care and procedures were performed in strict accordance with the guidelines and regulations of the Association for Assessment and Accreditation of Laboratory Animal Care International (AAALAC) under the permit number of SYXK-2011-0116. All experiments underwent scrutiny and approval by the Animal Care and Use Committee at the Shanghai Veterinary Research Institute, Chinese Academy of Agricultural Sciences, China.

### Virus strains, cell lines and antisera

The currently circulating PEDV strain FJzz1/2011 and classical PEDV strain HLJ/2011 were isolated and passaged in Vero cell lines supplemented with 5 μg/ml trypsin (Sigma-Aldrich, St. Louis, MO, USA). Cells were cultured in Dulbecco’s Modified Eagle’s Medium (DMEM, Gibco, Carlsbad, CA, USA) supplemented with 10% fetal bovine serum (Gibco, Carlsbad, CA, USA). The PEDV antisera were previously produced from pigs immunized with strain FJzz1/2011 and were preserved in our laboratory. The antisera used in this study were collected 1 day before and 14 and 21 days after immunization. In addition, the TGEV strain SH/2012, anti-TGEV N protein MAb and its hyperimmune antisera were obtained from SH/2012-immunized pigs and preserved in our laboratory.

### Development of MAbs

The PEDV strain FJzz1/2011 was purified and used as the antigen to immunize mice, and hybridomas secreting MAbs against PEDV were generated. To purify FJzz1/2011, 100 mL of virus suspension was collected and centrifuged at 5,000 × *g* for 30 min, 8,000 × *g* for 30 min, and 12,000 × *g* for 30 min to eliminate macromolecules. The supernatant was transferred to a new tube for ultracentrifugation at 30,000 × *g* for 1 h. The precipitate was resuspended in 1 mL of phosphate-buffered saline (PBS), followed by sucrose gradient centrifugation at 31,000 × *g* for 3 h. The purified PEDV suspension was mixed 1:1 with Freund’s complete adjuvant. Next, five BALB/c mice were intraperitoneally (ip) injected with the emulsified PEDV particles at a concentration of 50 μg per mouse. The interval between each injection was two weeks. After the third injection, spleen cells were removed and fused with SP20 cells to form hybridomas under aseptic conditions. The hybridomas were selectively cultured in HAT medium and HT medium. Finally, the positive hybridomas were screened by IFA, where the monolayers of Vero cells were inoculated with the PEDV strain FJzz1/2011 at a multiplicity of infection (MOI) of 0.01. Eighteen hours after infection, the cell lines were fixed with 80% ice-cold ethanol and were used for the selection of hybridomas that could steadily secrete MAbs against PEDV. The purified stable monoclonal hybridomas were then injected into the peritoneal cavity of mice to produce ascites fluid.

### Production of recombinant PEDV N protein and N protein truncated constructs

The whole RNA of FJzz1/2011 was extracted and converted into complementary DNA (cDNA). The entire N gene of PEDV was amplified from the cDNA by PCR using primers (Table 1) containing BamH1 and Xho1 restriction enzyme sites. The following PCR steps were used: initial denaturation at 94 °C for 3 min, followed by 32 cycles of denaturation at 94 °C for 30 seconds, annealing at 56 °C for 30 seconds and elongation at 70 °C for 30 seconds, and a final extension at 70 °C for 10 min. The obtained N gene was processed by restriction endonucleases BamH1 and Xho1 and was cloned into the prokaryotic expression vector pGEX-6p-1. The recombinant plasmid was transformed into the *Escherichia coli* strain BL21 (Tiangen, Beijing, China) and was expressed at 37 °C for 5 h in the presence of 1 mM isopropyl β-D-thiogalactoside (IPTG) (Sigma-Aldrich, St. Louis, MO, USA). The recombinant PEDV N protein was obtained and analyzed by sodium dodecyl sulfate-polyacrylamide gel electrophoresis (SDS-PAGE). Using the same method, we obtained a series of recombinant GST fusion N protein-truncated constructs, including GST-N1, GST-N2, GST-N1-1-GST-N1-11, and GST-N2-1-GST-N2-11.

### ELISA

The GST fusion N protein was coated onto ELISA plates (Thermo Fisher Scientific, Waltham, MA, USA) at 0.5 μg/well, followed by overnight incubation at 4 °C. The ELISA plates were blocked with 5% skimmed milk in PBST (PBS plus 0.5% Tween 20) at 37 °C for 2 h. Thereafter, the plates were washed three times with PBST, and ascites fluids raised against PEDV were added as primary antibodies to each well at a dilution of 1:200 at 37 °C for 1 h. The plates were washed again with PBST three times, and HRP conjugated goat anti-mouse IgG H&L (1/10,000 dilution) (Jackson ImmunoResearch, West Grove, PA, USA) was added as a secondary antibody at 37 °C for 1 h. The plates were washed with PBST five times, and 3,3′,5,5′-tetramethylbenzidine (TMB) substrate (Amresco, Cleveland, OH, USA) was added to each well for 10 min at room temperature. Finally, 2 M H_2_SO_4_ was added to the plates to stop the reaction, and the absorbance value of each well at 450 nm was read. To map the epitopes of N protein, all other GST fusion proteins (GST-N1, GST-N2, GST-N1-1-GST-N1-11, and GST-N2-1-GST-N2-11) were coated onto ELISA plates and were detected using the MAbs PN-D4 or PN-D6 as the primary antibodies using the same method above.

### Western blotting

Western blotting was used to further confirm the minimal antigenic regions targeted by MAbs PN-D4 and PN-D6 and to test whether the identified epitopes on N protein caused the cross-reactivity between PEDV and TGEV. The expressed N protein-truncated constructs were separated by SDS-PAGE on 12% gels and were transferred onto nitrocellulose (NC) membranes. After blocking with 5% skimmed milk for 2 h at room temperature, the membranes were exposed to primary antibodies for 1 h at room temperature. The primary antibodies used in the epitope mapping were MAbs PN-D4 and PN-D6, which were diluted 1:500 (0.5 μg/ml). The PEDV pig antisera and TGEV pig antisera were diluted 1:2000 to be used as the primary antibodies to detect the cross-reactivity between PEDV and TGEV. After unbound primary antibodies were rinsed, the membranes were immersed into the HRP conjugated goat anti-mouse IgG H&L or goat anti-pig IgG H&L and incubated for 30 min at room temperature. Additionally, the positive GST fusion proteins on the membranes were detected by chemiluminescence.

### IFA

IFA was performed to screen for MAbs specific to the PEDV strain FJzz1/2011 and to detect whether MAbs PN-D4 and PN-D6 could recognize the PEDV strain HLJ/2011 and TGEV strain SH/2012. Monolayers of Vero cells inoculated with FJzz1/2011 or HLJ/2011 at a MOI of 0.01 or monolayers of ST cells inoculated with TGEV strain SH/2012 at a MOI of 0.01 were incubated at 37 °C. Twelve hours later, the cell lines were gently rinsed and fixed with 80% ice-cold ethanol overnight at 4 °C. MAbs PN-D4 and PN-D6 (1:200) were added to the cells as primary antibodies for 1 h at 37 °C. The cells were then rinsed with PBS three times, and fluorescein (FITC)-conjugated donkey anti-mouse IgG (H + L) antibody (Life Technologies, Carlsbad, CA, USA) diluted to 1:2000 was added to cells at 37 °C for 1 h. The cells were then rinsed three times with PBS and stained with 4′,6-diamidino-2-phenylindole (DAPI). After incubation at 37 °C for 5 min, the cells were rinsed five times and analyzed by epifluorescence microscopy.

### Bioinformatics analysis

To further investigate the conservation and specificity of the identified epitopes, we selected another 22 PEDV representative strains (Table 2) and 32 representative coronavirus strains (Table 3) within the subfamily *Coronavirinae* for multiple sequence alignment using MegAlign Clustal W method in DNASTAR Lasergene software.

### Software prediction

A few online tools were used to characterize the determined epitopes. The GeneSilico MetaDisorder server (http://iimcb.genesilico.pl/metadisorder/) was used to predict the presence of intrinsically disordered regions of epitope NEP-D4^70^. The Phyre2 server (http://www.sbg.bio.ic.ac.uk/phyre2/) was used to predict the 3D structure of PEDV N protein^71^. Nine templates in PDB were selected for construction by this program (c4ud1B, d2bxxa1, d1sska, d2geca1, c3hd4A, d2cjra1, d2ca1a1, d2ge7a1 and d2giba1). In addition, the PredictProtein server (https://www.predictprotein.org) was used to predict the disulfide bridges formed between amino acids.

### Limited proteolysis and freeze-thaw cycle analyses

The recombinant protein GST-NEP-D4 was purified using Glutathione Sepharose 4B (GE Healthcare, Little Chalfont, UK), and the protein concentration was calculated by the absorbance at 280 nm (NanoDrop, Thermo Fisher Scientific, Waltham, MA, USA). Next, 10 μg of purified GST-NEP-D4 was removed and digested by 1 μg of trypsin (Sigma-Aldrich, St. Louis, MO, USA) in 100 mM KCl and 20 mM HEPES (pH 7.4 and 37 °C). The reaction was stopped by the addition of 1/6 volume of 6 × SDS loading buffer and heating to 100 °C for 5 min. Another 10 μg of purified GST-NEP-D4 was frozen at −80 °C for an hour, followed by thawing at room temperature. Finally, the samples treated with trypsin and freeze-thaw cycles were analyzed by Western blotting with MAb PN-D4 or an Anti-GST antibody, respectively, using the same methods described above.

## Additional Information

**How to cite this article**: Wang, K. *et al*. The Identification and Characterization of Two Novel Epitopes on the Nucleocapsid Protein of the Porcine Epidemic Diarrhea Virus. *Sci. Rep.* **6**, 39010; doi: 10.1038/srep39010 (2016).

**Publisher's note:** Springer Nature remains neutral with regard to jurisdictional claims in published maps and institutional affiliations.

## Figures and Tables

**Table 1:** Primers designed to amplify the N gene and its truncations.

Segments		Sequences (5′-3′)	Positions (Amino Acid)
N1-1	N1-1U	CGGGATCCATGGCTTCTGTCAGCTTTCAGG	1–150
	N1-1L	GCCCTCGAGCCTGCTACGTGAATTTGCACG	
N1-2	N1-2U	CGGGATCCATGGCTTCTGTCAGCTTTCAGG	1–140
	N1-2L	GCCCTCGAGAGGTGTGTTAGGTTCAACAATC	
N1-3	N1-3U	CGGGATCCATGGCTTCTGTCAGCTTTCAGG	1–139
	N1-3L	GCCCTCGAGTGTGTTAGGTTCAACAATCTC	
N1-4	N1-4U	CGGGATCCATGGCTTCTGTCAGCTTTCAGG	1–136
	N1-4L	GCCCTCGAGTTCAACAATCTCAACTACGCT	
N1-5	N1-5U	CGGGATCCATGGCTTCTGTCAGCTTTCAGG	1–133
	N1-5L	GCCCTCGAGCTCAACTACGCTGGGAAGC	
N1-6	N1-6U	CGGGATCCGTGCCATTATCCCTCTAT	1–130
	N1-6L	GCCCTCGAGGCTGGGAAGCTGTTGAGAGAAA	
N1-7	N1-7U	CGGGATCCGTGCCATTATCCCTCTAT	14–133
	N1-7L	GCCCTCGAGCTCAACTACGCTGGGAAGC	
N1-8	N1-8U	CGGGATCCGTGCCATTATCCCTCTAT	14–132
	N1-8L	GCCCTCGAGAACTACGCTGGGAAGCTGT	
N1-9	N1-9U	CGGGATCCTCCCTCTATGCCCCTCTT	17–133
	N1-9L	GCCCTCGAGCTCAACTACGCTGGGAAGC	
N1-10	N1-10U	CGGGATCCCTCTATGCCCCTCTTAGGGT	18–133
	N1-10L	GCCCTCGAGCTCAACTACGCTGGGAAGC	
N1-11	N1-11U	CGGGATCCTATGCCCCTCTTAGGGTTAC	19–133
	N1-11L	GCCCTCGAGCTCAACTACACTGGGGAGC	
N2-1	N2-1U	CGGGATCCAAGCTTAAGCAACAGCAGA	241–442
	N2-1L	GCCCTCGAGTTAATTTCCTGTATCGAAGAT	
N2-2	N2-2U	CGGGATCCAAGCTTAAGCAACAGCAGA	241–341
	N2-2L	GCCCTCGAG ACGAACAGCCACATTACC	
N2-3	N2-3U	CGGGATCCAAGCTTAAGCAACAGCAGA	241–308
	N2-3L	GCCCTCGAGTTCCGCATCTCCAAAAT	
N2-4	N2-4U	GATCCAAGCTTAAGCAACAGCAGAAGCCCAAACAGGAAAGGTCTGACAGCAGCGGCAAAAATACACCTAAGAAGC	241–263
	N2-4L	TCGAGCTTCTTAGGTGTATTTTTGCCGCTGCTGTCAGACCTTTCCTGTTTGGGCTTCTGCTGTTGCTTAAGCTTG	
N2-5	N2-5U	GATCCCAACAGCAGAAGCCCAAACAGGAAAGGTCTGACAGCAGCGGCAAAAATACACCTAAGAAGC	244–263
	N2-5L	TCGAGCTTCTTAGGTGTATTTTTGCCGCTGCTGTCAGACCTTTCCTGTTTGGGCTTCTGCTGTTGG	
N2-6	N2-6U	GATCCCAGGAAAGGTCTGACAGCAGCGGCAAAAATACACCTAAGAAGC	250–263
	N2-6L	TCGAGCTTCTTAGGTGTATTTTTGCCGCTGCTGTCAGACCTTTCCTGG	
N2-7	N2-7U	GATCC CAGGAAAGGTCTGACAGCAGCGGCAAAAATACACCTAAGC	250–262
	N2-7L	TCGAG CTTAGGTGTATTTTTGCCGCTGCTGTCAGACCTTTCCTGG	
N2-8	N2-8U	GATCCCAGGAAAGGTCTGACAGCAGCGGCAAAAATACACCTC	250–261
	N2-8L	TCGAGAGGTGTATTTTTGCCGCTGCTGTCAGACCTTTCCTGG	
N2-9	N2-9U	GATCC GAAAGGTCTGACAGCAGCGGCAAAAATACACCTAAGC	251–262
	N2-9L	TCGAG CTTAGGTGTATTTTTGCCGCTGCTGTCAGACCTTTCG	
N2-10	N2-10U	GATCC AGGTCTGACAGCAGCGGCAAAAATACACCTAAGC	252–262
	N2-10L	TCGAG CTTAGGTGTATTTTTGCCGCTGCTGTCAGACCTG	
N2-11	N2-11U	GATCC TCTGACAGCAGCGGCAAAAATACACCTAAGC	253–262
	N2-11L	TCGAG CTTAGGTGTATTTTTGCCGCTGCTGTCAGAG	

**Table 2:** PEDV strains used to align the sequences of NEP-D4 and NEP-D6.

Strains	Country	Collection Date	Accession Number	Lengths of N protein (Amino Acid)
FJzz1/2011	China	2011	Not submitted	441
ZJCZ4/2012	China	2011	JX524137	441
BJ-2011-1	China	2011	JN825712	441
CH-ZMDZY/11	China	2011	KC196276	441
CHGD-01	China	2011	JX261936	441
GD-B	China	2012	JX088695	441
IA1	USA	2013	KF468753	441
CH-YNKM-8	China	2013	KF761675	441
ISU13-22038-IA-homogenate	USA	2013	KF650373	441
13-019349	USA	2013	KF267450	441
MN	USA	2013	KF468752	441
NPL-PEDv	USA	2013	KJ778615	441
USA/Iowa/18984/2013	USA	2013	KF804028	441
OH851	USA	2014	KJ399978	441
KNU-1305	Korea	2014	KJ662670	441
KNU-1406-1	Korea	2014	KM403155	441
K14JB01	Korea	2014	KJ623926	441
KNU-141112-feces	Korea	2014	KR873431	441
KUIDL-PED-2014-001	Korea	2014	KJ588064	441
CV777	Belgium	1978	AF353511	441
HLJ/2011	China	2011	Not submitted	441
CH/S	China	1986	JN547228	441
DR13	Korea	1999	JQ023161	441

**Table 3:** Coronavirus strains used to align the sequences of NEP-D4 and NEP-D6.

Strains	Country	Collection Date	Accession Number	Lengths of N protein (Amino Acid)
TGEV-SH/2012	USA	2012	Not submitted	382
TGEV-PUR46-MAD	USA	2001	AJ271965	382
TGEV-Purdue	USA	1952	DQ811789	382
TGEV-Miller-M6	USA	2006	DQ811785	382
TGEV-KT2	Korea	2012	JQ693059	382
TGEV-HX	China	2012	KC962433	382
HCoV-229E-933-40	USA	1993	KF514433	389
HCoV-229E-0349	Netherlands	2010	JX503060	389
HCoV-NL63/DEN/2005/193	USA	2005	JQ765568	377
HCoV-NL63/DEN/2009/20	USA	2009	JQ765567	377
PRCV-ISU-1	USA	2006	DQ811787	382
PRCV-OH7269	USA	2014	KR270796	382
FIPV-DF-2	Hungary	2012	JQ408981	377
FIPV-79-1146	USA	1979	AY994055	377
SARS-MA-15	USA	2006	DQ497008	422
SARS-TW1	Taiwan	2003	AY291451	422
MERS-CoV-Riyadh_2_2012	Saudi Arabia	2012	KF600652	413
MERS-England/3/2013	UK	2013	KM21027	413
MHV-A59	USA	2004	AY700211	454
MHV-JHM	USA	2013	AC_000192	455
IBV-Georgia	USA	1998	GQ504722	409
IBV-Mass41	USA	1985	FJ904723	409
PDCoV-Illinois121	USA	2014	KJ481931	342
PDCoV/USA/Ohio137	USA	2014	KJ601780	342
PDCoV/CHJXNI2	China	2015	KR131621	342
